# Simulation-Based Education and Nursing Student Learning Motivations: A Scoping Review

**DOI:** 10.7759/cureus.84375

**Published:** 2025-05-18

**Authors:** Keisuke Nojima, Ryosuke Yoshida, Seiichi Sato, Tomohide Fukuda, Kanae Kakinuma

**Affiliations:** 1 Faculty of Nursing, Kyoto Tachibana University, Kyoto, JPN; 2 Department of Nursing, University of Osaka Hospital, Suita, JPN; 3 Faculty of Nursing, Musashino University, Koto, JPN; 4 Department of Nursing, International University of Health and Welfare, Otawara, JPN

**Keywords:** learning motivations, nursing education, nursing student, simulation-based education, simulation-based learning

## Abstract

This scoping review aimed to explore how simulation-based learning (SBL) influences learning motivation among undergraduate nursing students and to clarify the factors within SBL that contribute to increased motivation.

A scoping review methodology was employed following the Joanna Briggs Institute (JBI) guidelines and Preferred Reporting Items for Systematic Reviews and Meta-Analyses extension for Scoping Reviews (PRISMA-ScR) reporting standards. Literature published between January 2014 and November 2024 was systematically searched across MEDLINE, Cumulative Index to Nursing and Allied Health Literature (CINAHL), Education Resources Information Center (ERIC), and PubMed using relevant keywords based on the population-concept-context (PCC) framework. Studies were selected and reviewed by multiple independent reviewers, and data were extracted and analyzed thematically using an inductive approach.

Twenty-one studies were included. SBL was found to positively influence nursing students' intrinsic motivation, self-efficacy, critical thinking, and academic engagement. Simulation modalities included high-fidelity simulation, virtual reality, video-based learning, and standardized patients. Key motivational factors identified were realism in clinical scenarios, opportunities for repetition and reflection, structured debriefing, and emotionally engaging learning environments. Integration with problem-based learning and immersive technologies further enhanced learner engagement. However, challenges such as high implementation costs, the need for trained facilitators, and limited adaptability to diverse learner needs were noted.

This review offers insight into how SBL strategies can be optimized to support both cognitive and emotional aspects of learning motivation in nursing education and contributes to the literature by systematically mapping motivational factors across diverse simulation modalities.

## Introduction and background

In the rapidly evolving landscape of healthcare, the quality of nursing education is of paramount importance, as a well-educated, professional nursing workforce is essential to achieving good healthcare outcomes [1]. Nursing students must acquire both technical skills and theoretical knowledge as well as the motivation to engage actively in their learning and professional development [2,3]. Learning motivation is a critical factor in achieving academic success and ensuring the provision of competent and compassionate patient care.

Motivation in learning is a complex psychological state that consists of the desire to learn and a willingness to complete activities related to that learning [4]. It can be broadly categorized into intrinsic and extrinsic forms. Intrinsic motivation refers to the internal desire to learn for personal growth and satisfaction, while extrinsic motivation is driven by external rewards or outcomes, such as grades or recognition. Kolb's experiential learning theory suggests that individuals learn through a cyclical process of experience and reflection and that learners exhibit distinct styles such as diverging, assimilating, converging, and accommodating. These styles may influence how students engage with simulation-based learning (SBL). While not directly assessing motivation, it found that learning styles were associated with study duration and academic success, suggesting that aligning educational strategies with learning preferences could potentially support better engagement and outcomes [5].

The relationship between motivation and self-efficacy is reported to exist. Bandura's self-efficacy theory provides a robust framework for understanding motivation and behavior across various domains, emphasizing its influence on cognitive processes and motivation throughout the lifespan, including memory and learning [6]. In the context of nursing education, self-efficacy serves as a crucial predictor of students' performance, highlighting the importance of fostering confidence as a foundation for competency development in clinical practice [7].

Among the various educational strategies employed in nursing education, SBL has emerged as a transformative method that bridges the gap between classroom instruction and clinical practice [8]. It involves the use of realistic scenarios, technologies, and environments to replicate the complexities of healthcare settings. It enables students to practice clinical decision-making, teamwork, and hands-on skills in a safe, controlled environment, free from the risks of actual patient care. The SBL concept has been widely acknowledged for its potential to enhance cognitive, psychomotor, and affective learning domains [8,9]. Effective educational strategies, such as SBL, often aim to cultivate intrinsic motivation while aligning extrinsic incentives with meaningful learning goals. Particularly, its role in fostering learning motivation has become a subject of increasing academic and practical interest [10]. 

One of the key advantages of SBL is its ability to offer immediate feedback and opportunities for reflection. Debriefing sessions, which are integral to simulation exercises, allow students to analyze their performance, identify areas for improvement, and reinforce their strengths. This process enhances knowledge and technical skills, as well as confidence and self-efficacy, and helps maintain motivation for learning [8,11]. By providing students with realistic and engaging experiences, SBL has the potential to increase intrinsic motivation, as students find value and relevance in their learning activities [12].

Another significant benefit of SBL is its ability to simulate high-pressure, real-world scenarios in a controlled setting. These simulations challenge students to apply their knowledge and skills under stress, preparing them for the realities of clinical practice [13,14]. The sense of accomplishment derived from successfully navigating these scenarios can further enhance learning motivation, as students recognize the tangible impact of their efforts on patient care outcomes [15].　

Despite its numerous advantages, the implementation of SBL also presents challenges. The cost of high-fidelity simulation (HFS) equipment and the need for skilled facilitators can be significant barriers for educational institutions [10]. Additionally, the effectiveness of SBL in enhancing learning motivation can vary depending on factors such as the design of the simulation, the quality of facilitation, and individual student characteristics [16]. These challenges highlight the need for ongoing research and innovation to optimize SBL practices and maximize their impact on nursing education [17].

This review explores the potential of SBL as a method for enhancing learning motivation among nursing students. It aims to provide a comprehensive understanding of the mechanisms through which SBL influences motivation, the factors that contribute to its effectiveness, and the implications for nursing education. By synthesizing insights from existing research, this study seeks to guide educators in designing simulation experiences that develop technical competencies and also inspire and sustain student motivation to excel in their academic and professional pursuits.

## Review

Review questions

What is the relationship between simulation education and nursing students' motivation to learn? What factors in simulation education motivate nursing students to learn?

Methods

This scoping review was conducted according to the Joanna Briggs Institute (JBI) Scoping Review (ScR) methodology [18]. The review process is reported using the Preferred Reporting Items for Systematic Reviews and Meta-Analyses extension for Scoping Reviews (PRISMA-ScR) checklist [19].

Eligibility criteria

This scoping review applied inclusion criteria based on the population-concept-context (PCC) framework, as recommended by the JBI. The population included undergraduate nursing students at any level of study who were engaged in basic nursing education. The concept focused on simulation-based education and its influence on learning motivation, encompassing various simulation modalities such as HFS, virtual simulation, and the use of standardized patients. The context covered educational settings where SBL is implemented, including universities, clinical training environments, and online platforms across different cultural and geographical regions. Studies were eligible if they used quantitative designs (e.g., experimental, quasi-experimental, cross-sectional), qualitative methodologies (e.g., grounded theory, phenomenology), or systematic reviews, provided they contributed to understanding the relationship between simulation-based education and learning motivation. Opinion pieces and studies lacking methodological rigor were excluded. To ensure the relevance of the findings, only studies published between January 2014 and November 2024 were included.

Information sources

The databases searched were MEDLINE, Cumulative Index to Nursing and Allied Health Literature (CINAHL), Education Resources Information Center (ERIC), and PubMed. The search period was from January 2014 to November 2024.

Search strategy

Search terms were selected according to the PCC framework and included the following: “nursing student” AND (“Motivation” OR “Learning motivation” OR “Academic motivation”) AND (“simulation-based education” OR “simulation-based learning”). Boolean operators and wildcards were used to ensure comprehensive retrieval.

Study of the evidence selection

**Figure 1:**
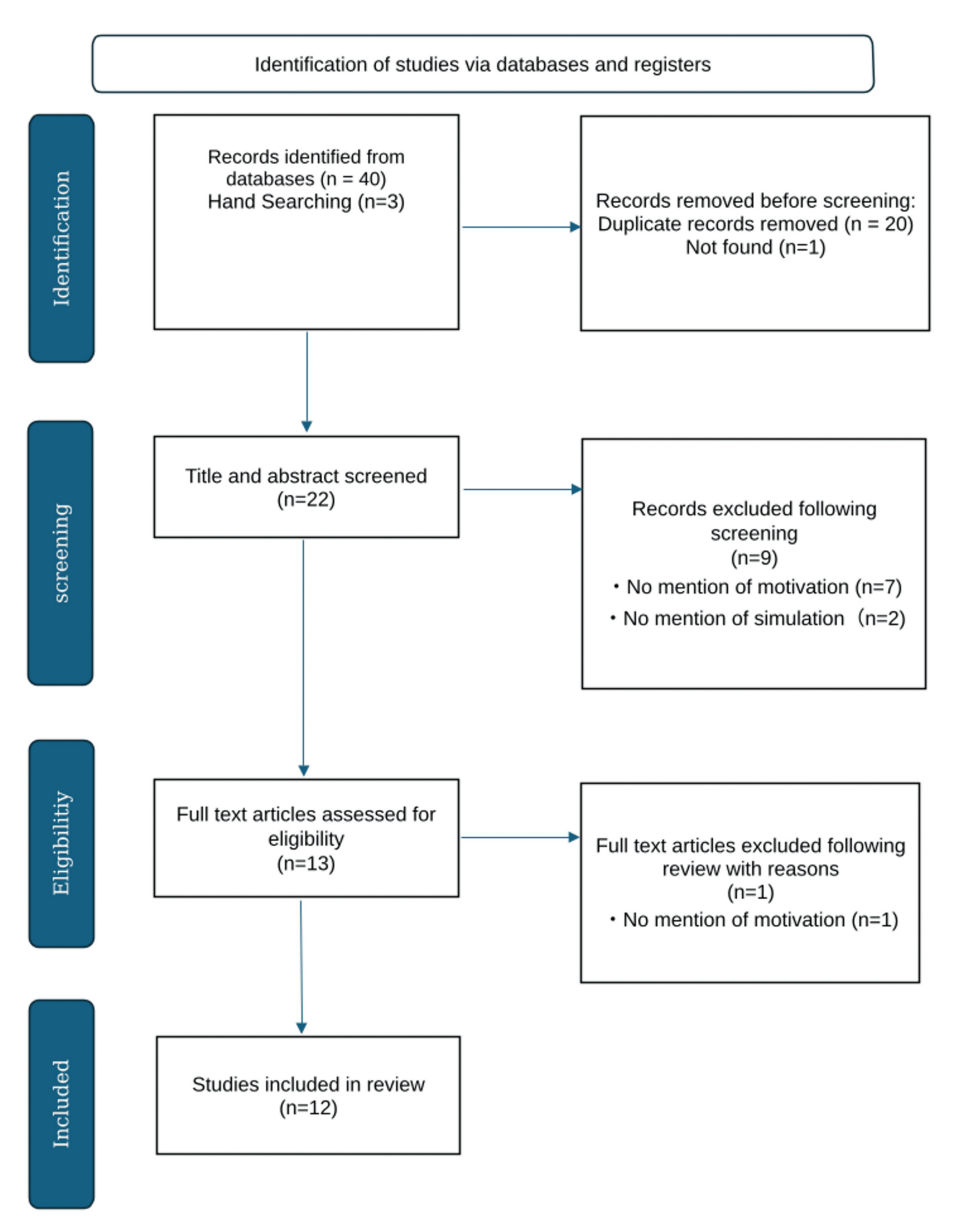
PRISMA-ScR flow diagram PRISMA-ScR: Preferred Reporting Items for Systematic Reviews and Meta-Analyses extension for Scoping Reviews

All identified citations were imported into EndNote (Clarivate, London, United Kingdom), and duplicates were removed. Two independent reviewers (KN, RY) screened titles and abstracts against the inclusion criteria. Full texts of potentially eligible studies were then reviewed. Discrepancies were resolved through discussion or with a third reviewer (TF or SS). The selection process is illustrated in a PRISMA-ScR flow diagram (Figure 1).

Data extraction

Data extraction was conducted independently by three reviewers (KN, RY, SS) using a standardized extraction tool. Extracted information included study characteristics (e.g., year, country, design, population), types of simulation modalities, and motivational outcomes. Thematic synthesis was used to identify key patterns and variations across studies. Disagreements were resolved within the research team and verified by additional reviewers (TF, KK).

Synthesis of results

Extracted data were organized into thematic categories based on recurring concepts and findings. A narrative synthesis was used to explore how simulation-based education influenced learning motivation among nursing students and which simulation components were most impactful.

Results

A total of 43 citations were identified in the database searches and reduced to 22 unique citations after the removal of duplicates. Based on the inclusion criteria, titles and abstracts were assessed for relevance, resulting in 13 citations. Full texts were analyzed, and one citation was excluded (Figure 1).

Characteristics of the included articles 

Geographical Distribution

The studies reflect a global interest in simulation-based education, with research conducted in North America [20,21], Europe [22], Asia [23,24], the Middle East [25], and South America [26]. Systematic reviews, such as [27], synthesized findings across multiple regions, emphasizing the global applicability.

Research Methodologies

Methodologies included systematic reviews [20,21], mixed-methods research [28,29], quantitative designs like randomized trials [25], and qualitative approaches [22,30]. These methods provided comprehensive insights into the educational impacts of simulation.

Study Populations

The primary focus was on undergraduate nursing students, with some studies including midwifery students [20]. Sample sizes ranged from small qualitative cohorts [22] to large-scale meta-analyses [21,27].

Data Collection Tools

Data collection included a standardized survey assessing learning motivation and performance [28]. The Self-Learning Methodology in Simulated Environments (MAES©) assessing self-directed learning [31], qualitative interviews [22], and clinical judgment scores [25] were used.

Simulation Modalities and Focus

Modalities included HFS [25], immersive virtual reality (VR) [29], standardized patients [21], video-based simulations [28], and problem-based learning (PBL) integration [23,24], each addressing specific educational goals.

Targeted Learning Outcomes

Key outcomes included enhanced motivation [26,28,30], critical thinking and clinical judgment [25,31], academic performance [28], teamwork [24], and self-directed learning [31], highlighting the comprehensive educational benefits of the simulation.

Factors motivating nursing students to learn in simulation education

SBL and Motivation: General Findings

SBL has proven to be a transformative tool in nursing education, effectively fostering intrinsic motivation and self-efficacy. Koçan et al. demonstrated that video-based simulations significantly enhanced student academic performance and motivation. Participants expressed higher levels of preparedness and confidence in applying theoretical knowledge to practical situations [28]. Further, Oh et al. conducted a meta-analysis revealing that simulations involving standardized patients led to notable improvements in communication skills and self-efficacy [21]. These findings emphasize the broad applicability of SBL in improving core competencies, thus making it critical in nursing curricula. Recent studies further substantiate these insights. Henrique-Sanches et al. conducted a scoping review to explore the implications of clinical simulations on learning motivation, finding that a variety of simulation methods consistently boosted student engagement and facilitated the integration of theoretical knowledge into clinical practice [26]. The review emphasized that well-structured simulations are highly useful in creating meaningful learning experiences that drive student motivation and academic success. Additionally, a study employing the MAES© methodology investigated the impact of self-directed learning with simulation on critical thinking and motivation among nursing students [31]. The pre-post intervention study revealed substantial improvements in students' ability to engage critically with clinical scenarios and maintain high levels of motivation. This underscores the effectiveness of combining self-directed learning with simulation techniques to foster not only cognitive development but also sustained engagement in nursing education. Together, these findings reinforce the role of SBL as a dynamic and important approach for cultivating motivation, critical thinking, and core competencies among nursing students.

HFS: A Key Motivational Driver

HFS has been recognized for its ability to replicate complex clinical scenarios with high levels of realism, offering students opportunities to practice in safe yet challenging environments. Fawaz and Hamdan-Mansour reported that nursing students engaged in HFS showed greater motivation and confidence compared to those exposed to traditional educational methods [25]. The simulations there allowed students to tackle high-pressure scenarios with reduced anxiety, fostering the ability to think critically and make informed decisions. Similarly, Lee et al. demonstrated that VR-enhanced HFS improved student engagement and motivation by enabling realistic patient interactions [29]. The combination of emotional involvement and clinical accuracy in these simulations suggests that HFS can be a highly useful tool in preparing students for real-world challenges.

PBL Integrated With Simulation

The integration of PBL with simulation has been identified as a powerful strategy to increase student engagement, fostering self-directed learning. Roh and Kim found that combining these methodologies encouraged collaborative learning and intrinsic goal orientation among students [24]. Through active problem-solving within simulated environments, learners developed critical thinking and decision-making skills essential for clinical practice. Lee et al. further highlighted the benefits of PBL-integrated simulations in enhancing metacognition and teamwork [23]. By addressing complex clinical problems in a guided yet interactive manner, students gained a deeper understanding of theoretical concepts and their practical applications.

Immersive Technology: VR and Augmented Reality (AR)

Immersive technologies like VR and AR are revolutionizing nursing education by providing interactive and engaging learning environments. Park et al. systematically reviewed studies on VR/AR in nursing education and found significant improvements in knowledge acquisition, motivation, and self-efficacy [27]. The interactive nature of these technologies enables students to practice repeatedly in a controlled environment, reinforcing their skills and confidence. For instance, Lee et al. demonstrated that VR-based scenarios for mental health nursing significantly heightened student motivation and engagement by allowing them to manage complex patient cases in a safe yet realistic manner [29]. These findings underline the potential of immersive technologies to transform traditional learning approaches.

Video-Based Simulation

Video-based simulation is an effective alternative to resource-intensive methodologies like HFS, particularly in settings with limited access to advanced technology. Koçan et al. reported that video simulations enhanced students' ability to retain theoretical knowledge and apply it in clinical settings [28]. This approach bridges the gap between theoretical and practical learning and complements other methodologies, such as VR, by providing foundational support. The flexibility and accessibility of video-based simulations make them an invaluable component of modern nursing education.

Clinical Skills Laboratories (CSLs): Enhancing Motivation Through Authentic Environments

CSLs are instrumental in creating authentic learning environments that encourage active participation and skill development. Haraldseid et al. emphasized that CSLs enable students to practice safely, effectively bridging the gap between classroom learning and real-world clinical application [22]. By providing realistic and interactive scenarios, CSLs motivate students to engage deeply with their studies, thereby enhancing their readiness for clinical practice.

Motivation in Specialized Scenarios: Mental Health and Neurological Nursing

Simulations focusing on specialized areas like mental health and neurological nursing offer unique opportunities to motivate students while addressing critical skill gaps. Lee et al. showed that HFS scenarios targeting complex neurological conditions significantly improved student metacognition and self-efficacy [23]. Similarly, Yahya et al. highlighted the effectiveness of mental health simulations in fostering empathy and intrinsic motivation [20]. By exposing students to high-stakes scenarios in a safe environment, these specialized simulations prepare them for complex clinical challenges while enhancing their motivation to learn.

Meta-Analysis of SBL Outcomes

Oh et al. conducted a comprehensive meta-analysis that demonstrated the consistent effectiveness of SBL in enhancing motivation, communication skills, and self-efficacy [21]. These results reaffirm the pivotal role of SBL in nursing education. Additionally, they expanded on these findings by exploring the longitudinal effects of simulation, showing sustained improvements in motivation and academic engagement over time. A summary of the 12 articles included in this review is displayed in Table 1.

**Table 1:** Data extraction table VR: virtual reality; SBL: simulation-based learning; MRPS: Motivation Resources and Problems Scale; PBL: problem-based learning; ANOVA: analysis of variance; HFS: high-fidelity simulation; CSL: Clinical skills laboratory; HMDs: head-mounted displays

No.	Author	Title	Year	Nation	Aim	Method	Population	Major findings
1	Yahya et al. [20]	Immersive simulation in nursing and midwifery education: a systematic review	2024	Morocco	To assess existing research evidence to identify the impact of immersive simulations on nursing and midwifery learning	Systematic reviews and meta-analyses	Nursing and midwifery students	It revealed that VR technology moderately enhances nursing students' engagement, motivation, and performance
2	Oh et al. [21]	The effects of simulation-based learning using standardized patients in nursing students: a meta-analysis	2015	South Korea	To evaluate the effect of SBL using standardized patients on cognitive, affective, and psychomotor domain outcomes of learning in nursing students	A meta-analysis	Nursing students	SBL using standardized patients might have a positive impact on self-efficacy and learning motivation that affects knowledge and clinical skill acquisition
3	Henrique-Sanches et al. [24]	Implications of clinical simulation in motivation for learning: scoping review	2024	Brazil	To identify, synthesize, and analyze the scientific knowledge produced regarding the implications of using clinical simulation for undergraduate nursing or medical students' motivation for learning	A scoping review	Nursing or medical students	The studies indicated the beneficial effects of clinical simulation on students' motivation, competencies, technical and non-technical skills, knowledge, belonging, autonomy, clinical judgment, critical and reflective thinking, self-efficacy and decreased anxiety, self-management, and improvements in learning and learning climate
4	Koçan et al. [28]	The effect of video-based simulation training on nursing students' motivation and academic achievement: a mixed study	2024	Turkey	To determine the effect of video-based simulation education on nursing students' motivation and academic achievement	Mixed model: a quasi-experimental method with a pretest-posttest control group and descriptive phenomenology	Nursing students	The increase in the posttest MRPS mean score of the intervention group was statistically significant (p=0.003). The mean posttest MRPS score in the intervention group was found to be significantly higher than that of the control group (p=0.002)
5	Lee et al. [23]	Effects of simulation with problem-based learning program on metacognition, team efficacy, and learning attitude in nursing students: nursing care with increased intracranial pressure patient	2017	South Korea	To identify the effects of simulation-based clinical practicum program with PBL on nursing students' metacognition, team efficacy, and learning attitude	A single-group pretest-posttest design	Nursing students	The results of the comparative analysis between pretest and posttest showed that there was a significant relationship between metacognition and motive for choosing a nursing major (p=0.040)
6	Roh and Kim [24]	Integrating problem-based learning and simulation: effects on student motivation and life skills	2015	South Korea	To assess learner motivation and life skills before and after taking a course involving PBL and simulation	A repeated-measures design	Nursing students	A repeated-measures ANOVA determined that the mean scores for total learner motivation scale (F=6.62; p=0.003) and total life skills scale (F= 8.89; p<0.001) differed significantly between time points
7	Fawaz and Hamdan-Mansour [25]	Impact of high-fidelity simulation on the development of clinical judgment and motivation among Lebanese nursing students	2016	Lebanon/Jordan	To examine the impact of using HFS on the development of clinical judgment and motivation among Lebanese nursing students	A posttest, quasi-experimental design	Nursing students	Nursing students exhibited significant improvement in clinical judgment and motivation due to exposure to HFS
8	Haraldseid et al. [22]	Nursing students' perceptions of factors influencing their learning environment in a clinical skills laboratory: a qualitative study	2015	Norway	To explore students' perceptions of their learning environment in a clinical skills laboratory and to increase the knowledge base for improving CSL learning conditions identifying the most important environmental factors according to the students	An exploratory qualitative method	Nursing students	The groups of 8-12 students in the CSL created an intimacy in the skills training that did not exist in lectures in an auditorium with 240 students. The positive experience that the students expressed could imply that they have a natural motivation to train and work in the CSL
9	Lee et al. [29]	Usability of mental illness simulation involving scenarios with patients with schizophrenia via immersive virtual reality: a mixed methods study	2020	South Korea	To evaluate the usefulness of VR simulation for mental health nursing education using videos recorded by 360-degree cameras and delivered via HMDs	Mixed-methods	Nursing students	Participants stressed that the high realism of the simulation increased their engagement in and motivation to learn about mental health nursing
10	Park et al. [27]	Effects of immersive technology-based education for undergraduate nursing students: systematic review and meta-analysis using the Grading of Recommendations, Assessment, Development, and Evaluation (GRADE) approach	2024	South Korea	To identify the contents of immersive technology–based education for undergraduate nursing students and evaluate the effectiveness of immersive technology compared to traditional teaching methods	Systematic reviews and meta-analyses	Nursing students	The outcomes included satisfaction, usability, a sense of realism, anxiety, knowledge, confidence, self-efficacy, performance, attitude, motivation, critical thinking, and clinical reasoning. Immersive technology can contribute to improving knowledge acquisition, confidence, and self-efficacy
11	Arizo-Luque et al. [31]	Does self-directed learning with simulation improve critical thinking and motivation of nursing students? A pre-post intervention study with the MAES© methodology	2022	Spain	To evaluate the learning motivation strategies of nursing students before and after the self-directed simulation training program (MAES©)	A multi-center, pre-post cross-sectional, and descriptive quantitative study	Nursing students	The students improved their levels of both motivation components (such as self-efficacy, strategy use, and self-regulation) and critical thinking components (such as personal characteristics, intellectual and cognitive abilities, interpersonal abilities and self-management, and technical abilities)
12	Rumahorbo et al. [30]	The effectiveness of human patient simulator on knowledge, motivation, and clinical competence of the student's nursing about diabetic ketoacidosis management	2018	Indonesia	To evaluate the effectiveness of human patient simulator training in improving knowledge, motivation, and clinical competence regarding diabetic ketoacidosis management	Quasi-experimental study	Nursing students	Human patient simulator training improved nursing students' knowledge, motivation, and clinical competence in managing diabetic ketoacidosis

Discussion

Designing SBL to Enhance Motivation

Effective instructional design is essential in SBL, as elements such as varying levels of difficulty, repetitive practice, interactivity, diverse learning strategies, individualized learning, and feedback have been shown to significantly improve learner competencies [32,33]. The findings of this review confirm that SBL can enhance nursing students' learning motivation. However, the implementation of simulation alone does not automatically yield motivational benefits; rather, its design and delivery play a pivotal role in shaping learning motivation.

HFS, by replicating realistic clinical settings, enables learners to experience authentic nursing scenarios [25]. However, the pursuit of realism alone does not necessarily lead to increased motivation. Instead, fostering a sense of responsibility, where students recognize that their decisions directly impact patient outcomes, appears to promote more proactive engagement. Therefore, HFS scenarios should be constructed to ensure students can experience the consequences of their own decisions and actions. This aligns with the understanding that decision-making processes influence learner engagement and motivation [34,35]. By incorporating divergent pathways and outcomes based on student choices, simulations can instill a heightened sense of responsibility and ownership over the learning process.

While high-fidelity technologies such as HFS and VR/AR enhance realism and immersion, it is critical to ensure they are not over-relied upon at the expense of fundamental clinical skills. For instance, securing sufficient time for repeated practice, a hallmark of SBL, is essential. In addition, deliberate integration of diverse instructional approaches such as task training, algorithm-based training, and situation-based training can support the development of basic skills such as physical assessment and communication. These structured modalities, when used in a balanced manner, help bridge the gap between technical sophistication and essential hands-on competencies.

The findings also underscore the importance of integrating various instructional tools and methodologies into SBL to influence student motivation. First, the integration of PBL with simulation fosters not only knowledge acquisition but also problem-solving skills directly relevant to clinical practice [24]. In this approach, students actively gather information, analyze situations, and engage in decision-making, which promotes deeper learning and sustained motivation. However, to avoid passivity during PBL-integrated simulations, it is essential to provide appropriately challenging tasks and structured feedback [36,37]. Pre- and post-simulation discussions among students can also enhance the learning process by encouraging reflection from diverse perspectives.

Although integrating PBL and fostering emotional engagement in simulations are effective strategies for increasing motivation, their implementation in large groups or resource-limited contexts presents practical challenges. To address this, adopting scalable models such as team-based learning (TBL) can facilitate collaborative engagement in larger cohorts. TBL allows for structured small-group discussions within large classrooms and can be adapted to include simulation components. Combined with rotation-based simulation sessions or blended learning formats, these models can maintain high levels of interaction and motivation while accommodating institutional constraints.

Moreover, the application of VR and AR in simulations has shown particular effectiveness in specialized fields such as psychiatric and neurological nursing [27]. These technologies allow students to repeatedly practice patient interactions in a safe and immersive environment. To maximize the motivational impact of VR/AR, however, it is not sufficient to emphasize visual realism alone; emotional engagement must also be fostered. Enhancing narrative structures and interactivity, such as branching scenarios driven by student choices, can deepen immersion and increase learning motivation.

Simulations employing standardized patients are also highly effective in improving interpersonal skills, particularly empathy and communication [21]. These simulations require students to respond in real-time to patient emotions and reactions, engaging emotional intelligence (EI), which is increasingly recognized as critical for nursing practice. Enhanced EI not only facilitates more effective relationships with patients, families, and colleagues [38] but also cultivates a sense of professional identity and long-term learning motivation.

Challenges in Implementing SBL to Enhance Motivation

Despite the benefits of SBL in enhancing learning motivation, its implementation is accompanied by several challenges. Notable barriers include high costs, the need for instructor expertise, the diversity of learner needs, and the lack of standardized evaluation measures.

One of the most pressing issues is the high cost associated with implementing HFS and VR/AR technologies [27]. These technologies require not only significant initial investment but also ongoing expenses for maintenance and software updates. For institutions with limited financial resources, these costs can be prohibitive. Thus, it is necessary to explore cost-effective alternatives, such as low-fidelity simulations or shared resource models across institutions.

Instructor competency is another critical factor influencing the success of simulation-based education [20]. In particular, the quality of debriefing sessions significantly impacts learning outcomes [29,39]. Poorly facilitated debriefings may prevent students from internalizing their experiences, reducing the educational effectiveness of simulations. Facilitators must be adequately trained in debriefing techniques, yet existing literature indicates variability in facilitation quality within nursing education [40].

Addressing learner diversity is also essential. The effectiveness of simulation varies depending on individual learning styles and motivational factors [31]. Students with high intrinsic motivation tend to prefer self-directed learning, while those relying on extrinsic motivation may benefit more from structured feedback and reward systems [41]. Barut et al. further support this view by showing that study preferences vary according to gender, academic year, and region, emphasizing the importance of adapting educational approaches to learner diversity [42]. Therefore, SBL programs should incorporate flexible designs that accommodate diverse learner needs.

Finally, the absence of standardized evaluation tools for assessing the outcomes of simulation education remains a significant challenge [21]. The current body of research employs varied measurement instruments, making cross-study comparisons difficult. Moving forward, the development of validated and comprehensive tools capable of assessing improvements in motivation, clinical judgment, and learning outcomes is necessary.

In summary, maximizing the effectiveness of simulation-based education requires addressing cost constraints, investing in instructor training, accommodating learner diversity, and establishing standardized evaluation frameworks. Strengthening facilitator training, particularly in debriefing techniques, can help ensure consistency and quality across institutions. Furthermore, future research should focus on developing reliable evaluation tools that not only measure motivation but also assess long-term retention and clinical application. Overcoming these challenges will enable SBL to fulfill its potential as a powerful and motivating educational strategy, thereby enhancing the overall quality of nursing education.

## Conclusions

This scoping review identified that SBL, when appropriately designed and implemented, contributes substantially to enhancing nursing students' motivation to learn. Across various modalities, including HFS, standardized patient encounters, video-based scenarios, and immersive technologies such as VR and AR, SBL was found to promote intrinsic motivation, self-efficacy, critical thinking, and academic engagement.

Key factors associated with increased learning motivation included the authenticity of clinical scenarios, opportunities for repetitive and self-directed practice, structured debriefing sessions that promote reflection, and emotionally engaging experiences. Notably, the integration of PBL within simulation, as well as the use of narrative-driven and interactive formats in virtual simulations, appeared particularly effective in sustaining motivation over time.

However, the review also highlighted challenges in the implementation of SBL. These included the high costs associated with advanced simulation technologies, variability in facilitator competency, limited adaptability to individual learner characteristics, and the lack of standardized tools for evaluating motivational outcomes. Addressing these challenges will require institutional commitment to faculty development, the adoption of scalable and context-appropriate simulation strategies, and the development of validated assessment instruments.

In conclusion, SBL represents a pedagogically robust strategy for fostering sustained motivation and competency development among undergraduate nursing students. To fully leverage its potential, simulation education should be designed not only to replicate clinical reality but also to actively engage learners cognitively, emotionally, and socially. Future research should further explore the mechanisms through which specific instructional designs and facilitation techniques influence motivation, with the aim of establishing best practices for simulation-based pedagogy in nursing education.
